# Supplementary material for: Health care expenditures among long-term survivors of pediatric solid tumors: Results from the French Childhood Cancer Survivor Study (FCCSS) and the French network of cancer registries (FRANCIM)
Source: PLoS One. 2022 May 26;17(5):e0267317. doi: 10.1371/journal.pone.0267317 (PMC9135272; doi:10.1371/journal.pone.0267317)
Supplement: S1 Table — (DOCX) [file pone.0267317.s001.docx]

Supplementary Table 1. Participating French administrative areas in FRANCIM.

| "Departement" (French administrative areas) | Year of Started | N | Already included in FCCSS |
| --- | --- | --- | --- |
| Ardennes | 1980 | 31 | 0 |
| Bas-Rhin | 1975 | 396 | 12 |
| Calvados | 1978 | 149 | 36 |
| Côte-d'Or | 1981 | 16 | 0 |
| Doubs | 1978 | 140 | 6 |
| Haut-Rhin | 1981 | 102 | 3 |
| Hérault | 1986 | 120 | 2 |
| Isère | 1979 | 217 | 7 |
| Lorrain | 1983 | 337 | 10 |
| Manche | 1994 | 59 | 6 |
| Martinique | 1981 | 16 | 6 |
| Somme | 1982 | 155 | 38 |
| Tarn | 1981 | 62 | 10 |
| Loire-Atlantique & Vendée | 1991 | 69 | 3 |
| Total | - | 1869 | 139 |
