# Supplementary material for: Health care expenditures among long-term survivors of pediatric solid tumors: Results from the French Childhood Cancer Survivor Study (FCCSS) and the French network of cancer registries (FRANCIM)
Source: PLoS One. 2022 May 26;17(5):e0267317. doi: 10.1371/journal.pone.0267317 (PMC9135272; doi:10.1371/journal.pone.0267317)
Supplement: S2 Table — (DOCX) [file pone.0267317.s002.docx]

| Supplementary Table 2. Survivors characteristics by Cohort | | |  |  |  |
| --- | --- | --- | --- | --- | --- |
|  | FCCSS | | French cancer registry | | Chi2 |
|  | n (%) | PY | n (%) | PY |  |
| Total | 3589 | 21247.6 | 1730 | 10286.0 |  |
| Sex |  |  |  |  |  |
| Man | 1972 (54.95%) | 11672.7 | 957 (55.32%) | 5679.1 | 0.80 |
| Women | 1617 (45.05%) | 9574.9 | 773 (44.68%) | 4606.9 |  |
| Year of diagnosis |  |  |  |  |  |
| <1980 | 926 (25.8%) | 5391.9 | 62 (3.58%) | 368.4 | <.0001 |
| 1980-1989 | 1292 (36%) | 7660.0 | 560 (32.37%) | 3314.5 |  |
| >=1990 | 1371 (38.2%) | 8195.7 | 1108 (64.05%) | 6603.1 |  |
| Age at first cancer |  |  |  |  |  |
| 0-1 | 781 (21.76%) | 4639.0 | 156 (9.02%) | 936.0 | <.0001 |
| 2-4 | 826 (23.01%) | 4899.9 | 208 (12.02%) | 1240.3 |  |
| 5-9 | 821 (22.88%) | 4842.0 | 267 (15.43%) | 1591.3 |  |
| 10-14 | 767 (21.37%) | 4535.6 | 363 (20.98%) | 2149.7 |  |
| ≥15 | 394 (10.98%) | 2331.1 | 736 (42.54%) | 4368.7 |  |
| Age at January 2011 |  |  |  |  |  |
| <20 | 392 (10.92%) | 2345.7 | 158 (9.13%) | 946.1 | <.0001 |
| 20-30 | 1311 (36.53%) | 7825.4 | 668 (38.61%) | 3987.4 |  |
| 31-40 | 1200 (33.44%) | 7110.7 | 678 (39.19%) | 4031.9 |  |
| 41-50 | 546 (15.21%) | 3147.4 | 207 (11.97%) | 1208.0 |  |
| >=51 | 140 (3.9%) | 818.4 | 19 (1.1%) | 112.6 |  |
| French geographical deprivation index |  |  |  |  |  |
| 1 Quintile | 833 (23.21%) | 4940.9 | 230 (13.29%) | 1372.0 | <.0001 |
| 2 Quintile | 742 (20.67%) | 4392.3 | 319 (18.44%) | 1901.0 |  |
| 3 Quintile | 648 (18.06%) | 3828.6 | 418 (24.16%) | 2493.7 |  |
| 4 Quintile | 667 (18.58%) | 3959.0 | 397 (22.95%) | 2361.5 |  |
| 5 Quintile | 699 (19.48%) | 4126.8 | 366 (21.16%) | 2157.8 |  |
| First primary cancer type |  |  |  |  |  |
| Other solid cancer | 549 (15.3%) | 1159.6 | 119 (6.88%) | 1676.9 | <.0001 |
| Kidney tumors | 471 (13.12%) | 3228.4 | 103 (5.95%) | 713.6 |  |
| Neuroblastoma | 631 (17.58%) | 2816.2 | 440 (25.43%) | 618.0 |  |
| Lymphoma | 414 (11.54%) | 3753.3 | 109 (6.3%) | 2597.3 |  |
| Soft tissue sarcomas | 325 (9.06%) | 2465.6 | 120 (6.94%) | 651.6 |  |
| Bone sarcomas | 466 (12.98%) | 1927.8 | 290 (16.76%) | 709.0 |  |
| Central nervous system tumor | 230 (6.41%) | 2708.7 | 159 (9.19%) | 1719.0 |  |
| Gonadal tumor | 36 (1%) | 1375.7 | 73 (4.22%) | 946.6 |  |
| Thyroid tumor | 269 (7.5%) | 212.7 | 36 (2.08%) | 438.0 |  |
| Retinoblastoma | 198 (5.52%) | 1599.5 | 281 (16.24%) | 216.0 |  |
| Status at December 2016 |  |  |  |  |  |
| Alive | 3462 (96.46%) | 20772.0 | 1690 (97.69%) | 10140.0 | 0.02 |
| Death | 127 (3.54%) | 475.6 | 40 (2.31%) | 146.0 |  |

PY: Person-years of follow-up
