# Supplementary material for: Health care expenditures among long-term survivors of pediatric solid tumors: Results from the French Childhood Cancer Survivor Study (FCCSS) and the French network of cancer registries (FRANCIM)
Source: PLoS One. 2022 May 26;17(5):e0267317. doi: 10.1371/journal.pone.0267317 (PMC9135272; doi:10.1371/journal.pone.0267317)
Supplement: S3 Table — (DOCX) [file pone.0267317.s003.docx]

| Supplementary Table 3. Health care expenditures by Sex | | |  |  |  |  |  |  |
| --- | --- | --- | --- | --- | --- | --- | --- | --- |
|  | Men | | | | Women | | | |
| Type of Expenditure | N° Patients (%) | N° Claims | Total Expenditures in Millions € (%) | Annual mean Per-Patient in € (SD) | N° Patients (%) | N° Claims | Total Expenditures in Millions € (%) | Annual mean Per-Patient in € (SD) |
| General practitioner visits | 2762 (94.3) | 66 396 | 1.4 (2.13) | 81 (131) | 2293 (95.94) | 77 711 | 1.6 (2.41) | 115 (133) |
| Other specialist visits | 2672 (91.23) | 60 268 | 2.2 (3.34) | 127 (435) | 2263 (94.69) | 101 730 | 3.2 (4.64) | 223 (438) |
| Physiotherapy visits | 1002 (34.21) | 55 174 | 0.9 (1.42) | 54 (298) | 1193 (49.92) | 68 654 | 1.2 (1.72) | 83 (341) |
| Nursing visits | 1711 (58.42) | 52 764 | 0.7 (1.07) | 41 (520) | 1808 (75.65) | 65 784 | 1.0 (1.52) | 73 (781) |
| Other health professionals visits | 267 (9.12) | 6 560 | 0.2 (0.32) | 12 (151) | 330 (13.81) | 5 988 | 0.2 (0.24) | 11 (112) |
| Pharmacy | 2742 (93.62) | 216 045 | 12.5 (18.86) | 719 (12906) | 2304 (96.4) | 286 827 | 8.8 (12.98) | 622 (2644) |
| Medical device | 2157 (73.64) | 25 558 | 4.6 (6.98) | 266 (1913) | 2066 (86.44) | 26 921 | 3.6 (5.25) | 252 (1650) |
| Laboratory Test | 2376 (81.12) | 35 703 | 0.9 (1.41) | 54 (348) | 2221 (92.93) | 65 543 | 1.6 (2.35) | 113 (228) |
| Technical medical procedures** | 2530 (86.38) | 21 283 | 1.1 (1.64) | 62 (218) | 2170 (90.79) | 27 300 | 1.5 (2.23) | 107 (351) |
| Transport | 895 (30.56) | 11 575 | 1.9 (2.89) | 110 (782) | 848 (35.48) | 13 029 | 2.5 (3.67) | 176 (1174) |
| Hospitalizations | 1717 (58.62) | 16 670 | 30.0 (45.16) | 1722 (11488) | 1733 (72.51) | 18 331 | 30.7 (45.06) | 2160 (16049) |
| Disability Benefits*** | 175 (5.97) | 7 223 | 4.2 (6.32) | 241 (1516) | 170 (7.11) | 6 640 | 3.7 (5.46) | 262 (1564) |
| Sick Leave | 1485 (50.7) | 17 832 | 5.0 (7.58) | 289 (1280) | 1246 (52.13) | 23 890 | 7.9 (11.62) | 557 (1953) |
| Others | 300 (10.24) | 1 786 | 0.6 (0.87) | 33 (1088) | 271 (11.34) | 1 656 | 0.6 (0.86) | 41 (975) |
| Total | 2929 | 594 837 | 66,3 | 3814 (19289) | 2390 | 790 004 | 68,2 | 4795 (18147) |

** Technical medical procedures includes expenditures mainly related to medical imaging techniques. *** Disability benefits includes all welfare payments or pensions made by the French Government to assistance people with disabilities.
