# Supplementary material for: Health care expenditures among long-term survivors of pediatric solid tumors: Results from the French Childhood Cancer Survivor Study (FCCSS) and the French network of cancer registries (FRANCIM)
Source: PLoS One. 2022 May 26;17(5):e0267317. doi: 10.1371/journal.pone.0267317 (PMC9135272; doi:10.1371/journal.pone.0267317)
Supplement: S4 Table — (DOCX) [file pone.0267317.s004.docx]

| Supplementary Table 4. Health care expenditures by Cohort | | |  |  |  |  |  |  |
| --- | --- | --- | --- | --- | --- | --- | --- | --- |
|  | FCCSS | | | | French Cancer Registry | | | |
| Type of Expenditure | N° Patients (%) | N° Claims | Total Expenditures in Millions € (%) | Annual mean Per-Patient in € (SD) | N° Patients (%) | N° Claims | Total Expenditures in Millions € (%) | Annual mean Per-Patient in € (SD) |
| General practitioner visits | 3396 (94.62) | 93 607 | 2.0 (2.07%) | 94 (132) | 1659 (95.9) | 50 500 | 1.0 (2.79%) | 102 (136) |
| Other specialist visits | 3318 (92.45) | 105 762 | 3.6 (3.68%) | 168 (476) | 1617 (93.47) | 56 236 | 1.8 (4.83%) | 176 (350) |
| Physiotherapy visits | 1441 (40.15) | 81 451 | 1.4 (1.45%) | 66 (323) | 754 (43.58) | 42 377 | 0.7 (1.88%) | 68 (310) |
| Nursing visits | 2337 (65.12) | 77 885 | 1.1 (1.15%) | 52 (615) | 1182 (68.32) | 40 663 | 0.6 (1.68%) | 61 (718) |
| Other health professionals visits | 426 (11.87) | 8 653 | 0.3 (0.27%) | 12 (130) | 171 (9.88) | 3 895 | 0.1 (0.3%) | 11 (143) |
| Pharmacy | 3398 (94.68) | 335 479 | 16.2 (16.65%) | 758 (11720) | 1648 (95.26) | 167 393 | 5.2 (13.89%) | 505 (2597) |
| Medical device | 2848 (79.35) | 36 957 | 5.9 (6.11%) | 278 (1847) | 1375 (79.48) | 15 522 | 2.3 (6.09%) | 221 (1697) |
| Laboratory Test | 3074 (85.65) | 69 462 | 1.7 (1.8%) | 82 (351) | 1523 (88.03) | 31 784 | 0.8 (2.1%) | 76 (158) |
| Technical medical procedures** | 3153 (87.85) | 32 862 | 1.8 (1.88%) | 86 (317) | 1547 (89.42) | 15 721 | 0.8 (2.1%) | 76 (208) |
| Transport | 1225 (34.13) | 17 742 | 3.3 (3.42%) | 156 (1037) | 518 (29.94) | 6 862 | 1.1 (2.95%) | 107 (844) |
| Hospitalizations | 2397 (66.79) | 25 748 | 44.6 (45.99%) | 2095 (11694) | 1053 (60.87) | 9 253 | 16.0 (42.82%) | 1556 (17186) |
| Disability Benefits*** | 238 (6.63) | 9 609 | 5.8 (5.93%) | 270 (1590) | 107 (6.18) | 4 254 | 2.2 (5.78%) | 210 (1423) |
| Sick Leave | 1789 (49.85) | 26 540 | 8.4 (8.62%) | 393 (1592) | 942 (54.45) | 15 182 | 4.6 (12.23%) | 444 (1686) |
| Others | 420 (11.7) | 2 503 | 1.0 (0.98%) | 45 (1240) | 151 (8.73) | 939 | 0.2 (0.56%) | 20 (358) |
| Total | 3589 | 924 260 | 97,1 | 4556 (18830) | 1730 | 460 581 | 37,4 | 3633 (18692) |

** Technical medical procedures includes expenditures mainly related to medical imaging techniques. *** Disability benefits includes all welfare payments or pensions made by the French Government to assistance people with disabilities.
