# Supplementary material for: Health care expenditures among long-term survivors of pediatric solid tumors: Results from the French Childhood Cancer Survivor Study (FCCSS) and the French network of cancer registries (FRANCIM)
Source: PLoS One. 2022 May 26;17(5):e0267317. doi: 10.1371/journal.pone.0267317 (PMC9135272; doi:10.1371/journal.pone.0267317)
Supplement: S5 Table — (DOCX) [file pone.0267317.s005.docx]

| Supplementary Table 5. Multivariate analysis for each type of expenditure | | | | | | | | | | | | | | | | |
| --- | --- | --- | --- | --- | --- | --- | --- | --- | --- | --- | --- | --- | --- | --- | --- | --- |
|  | General practitioner visits | Other specialist visits | Physiotherapy visits | Nursing visits | Other health professionals visits | Pharmacy | Medical device | Laboratory Test | Technical Medical Procedures** | | Transport | Hospitalizations | Disability Benefits*** | Sick Leave | Others |  |
| Intercept | 9.26 | -6.04 | -7.44 | -164.83 ** | -16.58 | 1.68 | 25.55 | 19.63 | -34.37 * | | -67.01 * | 9.24 | -1.85 | 18.77 | 81.49 |  |
| Women | 0.37 *** | 0.64 *** | 0.49 *** | 0.58 *** | -0.15 | 0.13 | 0.2 | 0.85 *** | 0.5 *** | | 0.69 *** | 0.24 ** | 0.18 | 0.7 *** | 0.1 |  |
| Age | 0.03 *** | 0.05 *** | 0.07 *** | 0.16 *** | 0.08 *** | 0.06 *** | 0.06 *** | 0.03 *** | 0.06 *** | | 0.09 *** | 0.04 ** | 0.18 *** | 0.03 *** | 0.06 |  |
| Year of Diagnosis | 0 | 0 | 0 | 0.08 ** | 0.01 | 0 | -0.01 | -0.01 | 0.02 * | | 0.03 * | 0 | 0 | -0.01 | -0.04 |  |
| Age at first cancer (Ref = 0-1) |  |  |  |  |  |  |  |  |  |  | |  |  |  |  |  |
| 2-4 | 0.1 ** | 0.1 | -0.15 | 0.2 | 0.16 | 0.88 ** | 0.14 | 0.25 *** | 0.08 | | 0.43 * | 0.19 | -0.12 | 0.25 ** | 0.85 ** |  |
| 5-9 | 0.1 * | 0.02 | -0.33 | -0.19 | 0.01 | 0.41 * | -0.23 | 0.21 ** | -0.08 | | -0.19 | 0.01 | -0.11 | 0.25 * | 0.62 |  |
| 10-14 | 0.11 | -0.04 | -0.5 * | -0.24 | -0.32 | 0.44 | -0.23 | 0.18 | 0.01 | | -0.73 ** | -0.05 | -0.26 | 0.45 *** | 1.34 ** |  |
| ≥15 | 0.03 | -0.12 | -0.63 ** | -0.98 | -1.11 ** | 0.03 | -0.82 * | 0.08 | -0.35 * | | -1.35 *** | -0.29 | 0.02 | 0.51 ** | 0.92 |  |
| French Index Deprivation | 0.08 *** | -0.01 | -0.01 | 0.16 ** | -0.03 | 0.01 | 0 | -0.01 | 0.03 | | 0.31 *** | 0.06 | 0.17 ** | 0.02 | 0.09 |  |
| First primary cancer type (Ref = Neuroblastoma) |  |  |  |  |  |  |  |  |  | |  |  |  |  |  |  |
| Kidney tumor | 0.04 | 0.11 | -0.12 | 0.12 | -0.5 | 0.99 ** | 0.1 | 0.23 ** | 0.14 | | 0.4 | 0.16 | -0.59 | 0.04 | 0.55 |  |
| Lymphoma | 0.17 *** | 0.21 *** | 0.31 | -0.93 *** | -0.25 | 0.23 | -0.49 | 0.21 ** | 0.19 * | | 0.1 | -0.01 | -0.22 | 0.09 | -0.15 |  |
| Soft tissue sarcoma | 0.18 *** | 0.12 | 0.58 ** | -0.58 | -0.2 | 0.08 | 0.36 | -0.07 | 0.32 *** | | 0.02 | 0.06 | -0.1 | 0.21 | 0.07 |  |
| Bone sarcoma | 0.33 *** | 0.13 | 0.96 *** | -0.86 ** | -0.65 | 0.23 | 1.63 *** | 0.05 | 0.31 *** | | 1.06 *** | 0.29 | 0.31 | 0.16 | 0.29 |  |
| Central nervous system tumor | 0.56 *** | 0.29 *** | 1.78 *** | 0.74 ** | 1.66 *** | 0.93 *** | 0.54 * | 0.23 ** | 0.55 *** | | 1.59 *** | 1.1 *** | 0.25 | -0.23 | 0.79 * |  |
| Gonadal tumor | -0.01 | 0.01 | 0.09 | -0.92 ** | -0.19 | 0.89 * | -0.72 | 0 | 0.37 ** | | 0.01 | -0.01 | -0.99 | -0.11 | 0.18 |  |
| Thyroid tumor | 0.3 *** | 0.27 | -0.13 | -1.66 *** | 1.28 * | 1.02 | -1.11 *** | 0.37 *** | 0.26 | | -0.6 | -0.74 ** | -1.55 * | 0.16 | -0.29 |  |
| Retinoblastoma | 0.16 ** | 0.07 | -0.1 | -0.07 | -1.18 *** | -0.08 | -0.24 | -0.14 | 0.2 | | 1.05 *** | 0.52 * | -0.21 | -0.38 * | 0.69 |  |
| Other solid cancer | 0.21 *** | 0.12 | 0.54 ** | -0.16 | 0.74 | 0.14 | -0.26 | 0.12 | 0.26 * | | 0.5 | 0.07 | -1.18 ** | 0.12 | 1.2 |  |
| FCCSS | -0.09 ** | -0.1 ** | -0.15 | -0.06 | 0.21 | 0.05 | -0.18 | -0.01 | 0.05 | | 0.33 ** | 0.25 * | -0.19 | -0.07 | 0.3 |  |

*** p<0.01, ** p<0.05, * p<0.10
