# Supplementary material for: Health care expenditures among long-term survivors of pediatric solid tumors: Results from the French Childhood Cancer Survivor Study (FCCSS) and the French network of cancer registries (FRANCIM)
Source: PLoS One. 2022 May 26;17(5):e0267317. doi: 10.1371/journal.pone.0267317 (PMC9135272; doi:10.1371/journal.pone.0267317)
Supplement: S6 Table — (DOCX) [file pone.0267317.s006.docx]

| Supplementary Table 6. Multivariate analysis for total health care expenditure by each type of cancer | | | | | | | | | | | |
| --- | --- | --- | --- | --- | --- | --- | --- | --- | --- | --- | --- |
|  | Kidney tumor (n = 668) | Neuroblastoma (n = 574) | Lymphoma (n = 1,071) | Soft tissue sarcoma (n = 523) | Bone sarcoma (n = 445) | Central nervous system tumor (n = 756) | Gonadal tumor (n = 389) | Thyroid tumor (n = 109) | Retinoblastoma (n = 305) | Other solid cancer (n = 479) |  |
| Intercept | -27.71 | -119.94 | 29.08 | 30.02 | -19.04 | 72.79 | -114.51 | -101.69 | 25.45 | 45.49 |  |
| Women | 0.16 | 0.11 | 0.73 *** | 0.69 *** | 0.37 ** | 0.13 | 0.7 ** | 0.08 | 0.25 | -0.11 |  |
| Age | 0.07 * | 0.12 *** | 0.05 ** | 0.05 | 0.05 ** | 0.02 | 0.1 *** | 0.05 | 0.06 * | 0.04 |  |
| Year of Diagnosis | 0.02 | 0.06 | -0.01 | -0.01 | 0.01 | -0.03 | 0.06 | 0.05 | -0.01 | -0.02 |  |
| Age at first cancer (Ref = ≥15) |  |  |  |  |  |  |  |  |  |  |  |
| 0-1 | -0.12 | 1.3 | 0.04 | 0.13 | -0.63 | 0.24 | 1.01 |  | 3.17 *** | -0.2 |  |
| 2-4 | 0.32 | 1.51 | 0.27 | 0.78 | -0.28 | 0.41 | 0.44 | -3.25 *** | 3 *** | 0.88 * |  |
| 5-9 | -0.21 | 1.44 | -0.05 | 0.35 | 0.36 | 0.36 | 0.8 | 0.33 | 2.27 *** | 0.34 |  |
| 10-14 | -0.79 | 0.78 | -0.12 | 0.45 | 0.15 | 0.69 ** | 0.34 | 0.59 | 3.36 *** | 0.42 |  |
| French Index Deprivation | -0.17 * | 0.18 ** | 0.13 *** | -0.05 | 0 | -0.01 | 0.31 ** | -0.04 | -0.12 | 0.05 |  |
| FCCSS | -0.12 | 0.48 * | 0.03 | 0.06 | -0.1 | -0.11 | 0.47 | 0.51 | 0.3 | 0.13 |  |

*** p<0.01, ** p<0.05, * p<0.10
