# Supplementary figures and images for: Health care expenditures among long-term survivors of pediatric solid tumors: Results from the French Childhood Cancer Survivor Study (FCCSS) and the French network of cancer registries (FRANCIM)
Source: PLoS One. 2022 May 26;17(5):e0267317. doi: 10.1371/journal.pone.0267317 (PMC9135272; doi:10.1371/journal.pone.0267317)

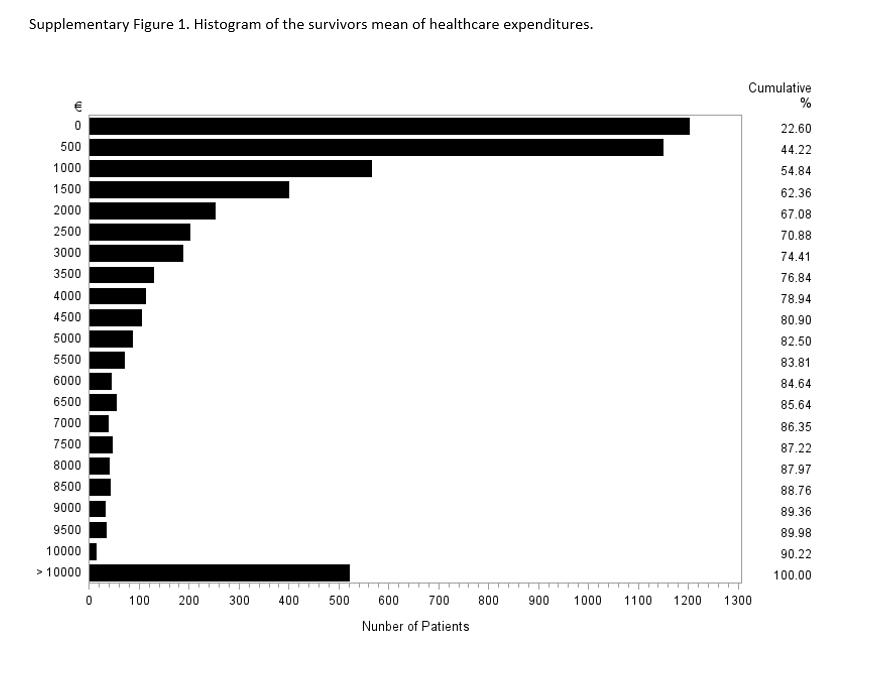

Supplement: S1 Fig — (TIF) [file pone.0267317.s007.tif]

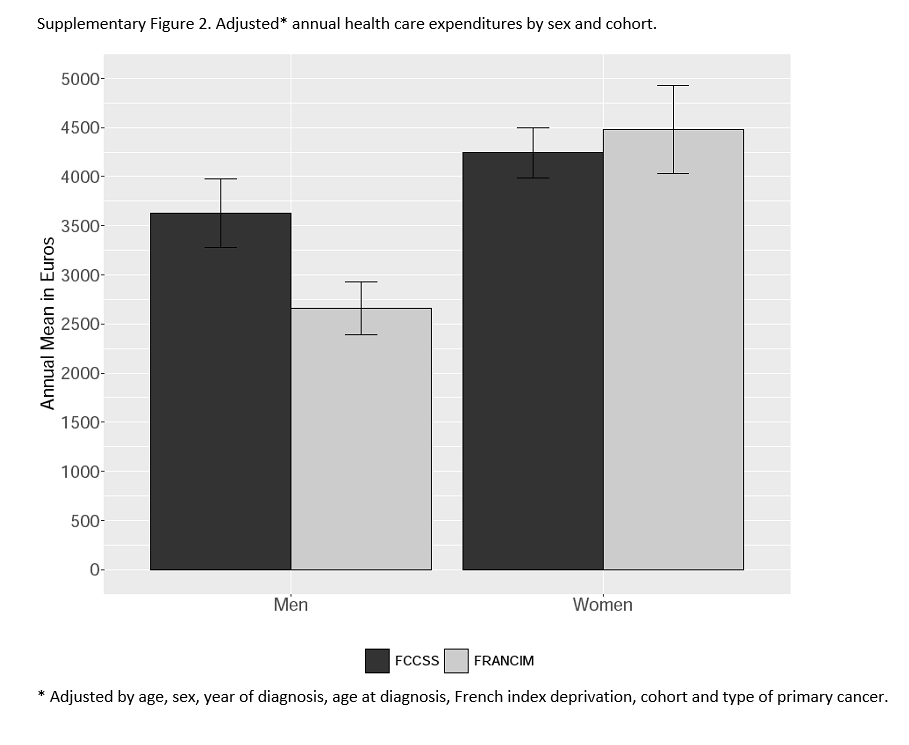

Supplement: S2 Fig — (TIF) [file pone.0267317.s008.tif]

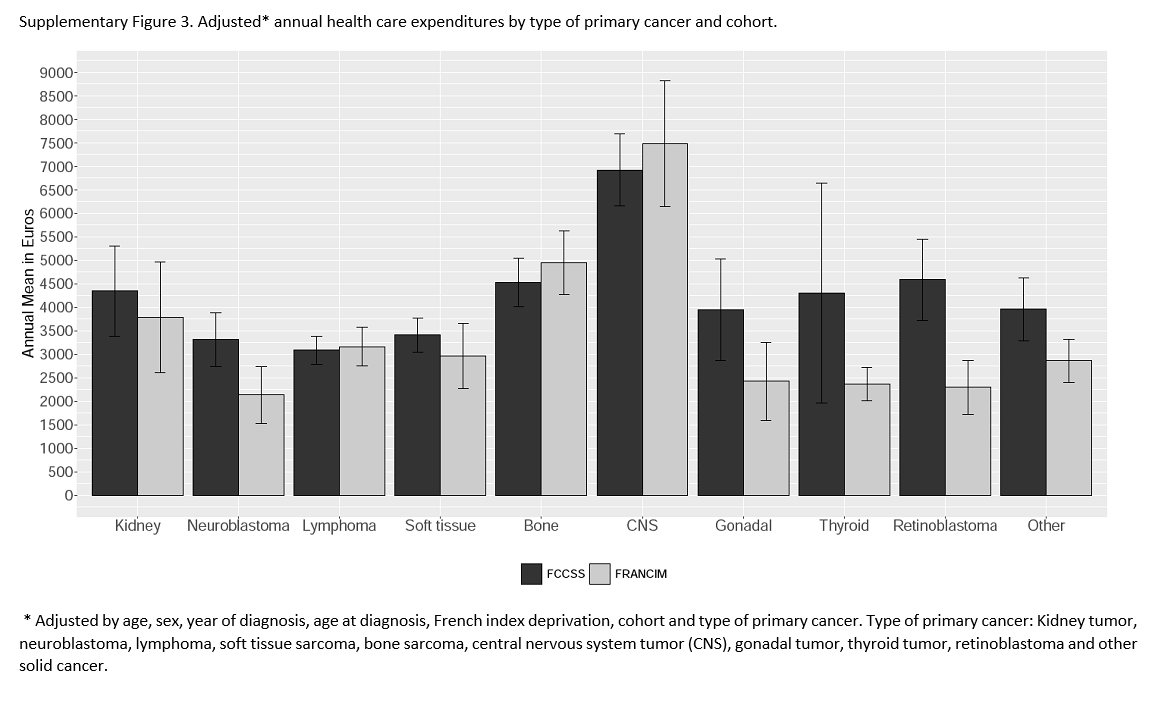

Supplement: S3 Fig — (TIF) [file pone.0267317.s009.tif]
