# Supplementary material for: Health care expenditures among long-term survivors of pediatric solid tumors: Results from the French Childhood Cancer Survivor Study (FCCSS) and the French network of cancer registries (FRANCIM)
Source: PLoS One. 2022 May 26;17(5):e0267317. doi: 10.1371/journal.pone.0267317 (PMC9135272; doi:10.1371/journal.pone.0267317)
Supplement: Attachment — Submitted filename: Response to Reviewers.docx [file pone.0267317.s011.docx]

Dear Editor-in-chief,

We are grateful to the editors and reviewers for their time and constructive comments on our manuscript. We have implemented their suggestions and answered their concerns which, we believe, allowed improving the manuscript. Changes in the initial version of the manuscript can be retrieved in the tracked change version. Below, we provide a point-by-point response explaining how we have addressed each of the editors or reviewers’ comments. We look forward to receiving your further evaluation of our manuscript.

Sincerely,

Florent de Vathaire, PhD

Head of the Radiation Epidemiology Group

Unit 1018 INSERM – CESP

Institut Gustave Roussy
39, rue Camille Desmoulins
94805 Villejuif, France
Tel: +33 1 42 11 54 57
Fax: +33 1 42 11 53 15
Email: [florent.devathaire@gustaveroussy.fr](mailto:florent.devathaire@gustaveroussy.fr)
Web: <https://www.gustaveroussy.fr/> - <https://cesp.inserm.fr/fr>

All page and line numbering below refers to the re-submitted clean copy, word document, which has been line numbered, before re-submission.

**Journal Requirements:**

**Please review your reference list to ensure that it is complete and correct. If you have cited papers that have been retracted, please include the rationale for doing so in the manuscript text, or remove these references and replace them with relevant current references. Any changes to the reference list should be mentioned in the rebuttal letter that accompanies your revised manuscript. If you need to cite a retracted article, indicate the article’s retracted status in the References list and also include a citation and full reference for the retraction notice.**

Our response: We thank the editor for your review. We have reviewed our reference list and have changed those that were found to be incorrect in order to fully meet PLOS ONE's style requirements.

Specific changes were made in the references:

Lines 401-403: “3.	Freyer DR. Transition of Care for Young Adult Survivors of Childhood and Adolescent Cancer: Rationale and Approaches. J Clin Oncol. 2010;28: 4810–4818. doi:10.1200/JCO.2009.23.4278.”

Lines 419-422: “9.	Berger C, Casagranda L, Faure-Conter C, Freycon C, Isfan F, Robles A, et al. Long-Term Follow-up Consultation After Childhood Cancer in the Rhône-Alpes Region of France: Feedback From Adult Survivors and Their General Practitioners. J Adolesc Young Adult Oncol. 2017;6: 524–534. doi:10.1089/jayao.2017.0019.”

Lines 471-473: “23.	Nipp RD, Kirchhoff AC, Fair D, Rabin J, Hyland KA, Kuhlthau K, et al. Financial Burden in Survivors of Childhood Cancer: A Report From the Childhood Cancer Survivor Study. J Clin Oncol. 2017;35: 3474–3481. doi:10.1200/JCO.2016.71.7066.”

Lines 509-512: “34.	van Dorp W, Haupt R, Anderson RA, Mulder RL, van den Heuvel-Eibrink MM, van Dulmen-den Broeder E, et al. Reproductive Function and Outcomes in Female Survivors of Childhood, Adolescent, and Young Adult Cancer: A Review. J Clin Oncol. 2018;36: 2169–2180. doi:10.1200/JCO.2017.76.3441.”

Lines 517-519: “36.	Hjern A, Lindblad F, Boman KK. Disability in Adult Survivors of Childhood Cancer: A Swedish National Cohort Study. J Clin Oncol. 2007;25: 5262–5266. doi:10.1200/JCO.2007.12.3802.”

**Additional Editor Comments:**

**Thank you for submitting your revised manuscript. Please consider the following additional comments in an additional revision and resubmission.**

**1. The wording of the revised title is awkward. Consider, "Healthcare expenditures among long-term survivors of pediatric solid tumors: Results from...."**

Our response: Taking up your suggestion, we have changed the title of the article to: “Healthcare expenditures among long-term survivors of pediatric solid tumors: Results from the French Childhood Cancer Survivor Study (FCCSS) and the French network of cancer registries (FRANCIM).

**2. The response to Comment 5 of Reviewer 1 should include the addition of this strength of the FCCSS study (long follow-up) to the Discussion.**

**Comment 5 – Reviewer 1. It took me some time to consider the inclusion of the diagnoses in the early years (they start in 1945) given the observation window for the outcome only starts in 2011, but I think this approach is strong as it produces estimates across the age spectrum.**

Our response: We have added more details regarding Comment 5 of Reviewer 1.

Lines 359-360: “Another strength is the inclusion period starting in 1945, which allows us to study variations in cost across the age spectrum.”

**3. The response to Comment 5 of Reviewer 2 is not clear and needs further explanation, including what is meant by the neuroblastoma group being "medically homogenous."**

Our response: We clarified this as follows:

Lines 190-192: “Neuroblastoma was chosen as the referent for type of primary cancer variable since was one of the larger group of cancer of same histology”.

**4. The term "paramedic" referring to costs needs clarification/definition.**

Our response: We thank you for pointing out that “paramedic” were not clear enough. We have changed the names of this category to “Other health professionals visits” which is a more accurate translation:

We have also added a footnote to better define what’s included in the Table 2:

† Other medical professional visits included expenditures related to visits to podiatrist, optometrists, speech therapist, and others.
